# Body temperature elevation during exercise is essential for activating the Akt signaling pathway in the skeletal muscle of type 2 diabetic rats

**DOI:** 10.1371/journal.pone.0205456

**Published:** 2018-10-10

**Authors:** Takamasa Tsuzuki, Toshinori Yoshihara, Noriko Ichinoseki-Sekine, Ryo Kakigi, Yuri Takamine, Hiroyuki Kobayashi, Hisashi Naito

**Affiliations:** 1 Graduate School of Health and Sports Science, Juntendo University, Chiba, Japan; 2 Faculty of Pharmacy, Meijo University, Aichi, Japan; 3 Faculty of Liberal Arts, The Open University of Japan, Chiba, Japan; 4 Faculty of Medicine, Juntendo University, Tokyo, Japan; 5 Department of General Medicine, Mito Medical Center, Tsukuba University Hospital, Ibaraki, Japan; Tohoku University, JAPAN

## Abstract

This study examined the effect of changes in body temperature during exercise on signal transduction-related glucose uptake in the skeletal muscle of type 2 diabetic rats. Otsuka Long-Evans Tokushima Fatty rats (25 weeks of age), which have type 2 diabetes, were divided into the following four weight-matched groups; control (CON, *n* = 6), exercised under warm temperature (WEx, *n* = 8), exercised under cold temperature (CEx, *n* = 8), and heat treatment (HT, *n* = 6). WEx and CEx animals were subjected to running on a treadmill at 20 m/min for 30 min under warm (25°C) or cold (4°C) temperature. HT animals were exposed to single heat treatment (40–41°C for 30 min) in a heat chamber. Rectal and muscle temperatures were measured immediately after exercise and heat treatment, and the gastrocnemius muscle was sampled under anesthesia. Rectal and muscle temperatures increased significantly in rats in the WEx and HT, but not the CEx, groups. The phosphorylation levels of Akt, AS160, and TBC1D1 (Thr590) were significantly higher in the WEx and HT groups than the CON group (*p* < 0.05). In contrast, the phosphorylation levels of AMP-activated protein kinase, ACC, and TBC1D1 (Ser660) were significantly higher in rats in the WEx and CEx groups than the CON group (*p* < 0.05) but did not differ significantly between rats in the WEx and CEx groups. Body temperature elevation by heat treatment did not activate the AMPK signaling. Our data suggest that body temperature elevation during exercise is essential for activating the Akt signaling pathway in the skeletal muscle of rats with type 2 diabetic rats.

## Introduction

Insulin resistance plays a key role in the development of lifestyle diseases, including obesity and type 2 diabetes. Skeletal muscle is the largest tissue in the body by mass and is the major site of glucose disposal [1]; it also plays a central role in regulating whole-body insulin resistance [2]. Because skeletal muscle insulin resistance is a predictor of type 2 diabetes, and maintenance of adequate muscle glucose disposal may help to prevent the disease [3], a therapeutic strategy for improving insulin resistance in the skeletal muscle of patients with obesity and type 2 diabetes is needed.

Regular exercise improves whole-body and peripheral tissue (*i*.*e*., skeletal muscle, liver, and adipose tissue) insulin resistance, however the factors that influence the beneficial effects of exercise are not fully understood. We reported previously that exercise training without body temperature elevation blunts the improvement in insulin resistance in type 2 diabetic rats [4], suggesting that body temperature elevation during exercise may be important for improving insulin resistance in type 2 diabetes. Although the mechanisms underlying this phenomenon are still unclear, it is likely that molecular factors affected by temperature alteration are involved.

The potential mechanisms by which exercise prevents obesity-induced insulin resistance include the transport of glucose into skeletal muscles [5]. Previous studies have reported that two distinct mechanisms—insulin signaling and exercise-induced mechanisms—are responsible for the stimulation of GLUT4 translocation to the plasma membrane and T-tube and glucose uptake [6]. A single bout of exercise activates multiple factors in skeletal muscle implicated in exercise-induced glucose transport, including AMP-activated protein kinase (AMPK), calcium/calmodulin-dependent protein kinase (CaMK), and atypical protein kinase C (aPKC) isoforms [6–8]. Akt is a key factor in insulin signaling [9, 10]; however, whether exercise induces Akt activity in the muscle has been controversial [11–14].

Previous studies have shown that the phosphorylation status of Akt at Ser473 is highly temperature-sensitive in vitro [15] and increases in a temperature-dependent manner in rat skeletal muscle [16]. Given these results, body temperature elevation would stimulate Akt phosphorylation in skeletal muscle. Body temperature is elevated during exercise under thermo-neutral conditions; however, the effects of body temperature elevation during exercise on Akt signaling in the skeletal muscle of type 2 diabetic rats are unclear. In addition, tre-2/USP6, BUB2, cdc16 domain family member 1 (TBC1D1), and Akt substrate of 160 kDa (AS160; also known as TBC1D4) are downstream proteins of Akt and AMPK [17, 18], and AS160 and TBC1D1 may serve as a point of convergence of insulin- and exercise-dependent signaling in the regulation of glucose uptake [6]. Therefore, it is possible that body temperature elevation during exercise enhances both the Akt and AMPK signaling pathways, which may be an effective strategy for glucose maintenance in type 2 diabetic individuals.

Herein, we examined whether the changes in body temperature during exercise affect signal transduction related glucose metabolism in the skeletal muscle of obese/type 2 diabetic rats. Based on our previous study [4], we hypothesize that suppression of body temperature elevation during exercise attenuates activation of Akt in the muscle, blunting the improvement in whole-body insulin resistance induced by exercise training in type 2 diabetic rats.

## Materials and methods

### Animals

All procedures were approved by the Juntendo University Animal Care and Use Committee and conducted according to the guiding principles for the Care and Use of Laboratory Animals set forth by the Physiological Society of Japan. Male Otsuka Long-Evans Tokushima Fatty (OLETF) rats were obtained from Japan SLC (Shizuoka, Japan). OLETF rats are a well-characterized animal model of human type 2 diabetes. OLETF rats exhibit hyperphagia and obesity beginning during early childhood and go on to develop insulin resistance and type 2 diabetes [19]. All animals were housed with 12:12-h light-dark cycle in an environment-controlled room (23 ± 1°C, 55 ± 5% relative humidity) and given standard rat chow and water *ad libitum*. At 25 weeks of age, OLETF rats (612.6 ± 30.7 g) were divided into four body weight-matched groups: control (CON; n = 6), exercised under warm temperature (WEx; n = 8), exercised under cold temperature (CEx; n = 8), and heat treatment (HT; n = 6).

### A single bout of exercise

WEx and CEx animals were familiarized with running on a motor-driven animal treadmill (KN-73; Natsume, Tokyo, Japan) for approximately 10–20 m/min for 5 min per day beginning 1 week before the experiment. After 5 h of fasting, they were subjected to run on the treadmill at 20 m/min without grade for 30 minutes in a climate-controlled room at 25°C or 4°C, respectively. Electric shocks were rarely used to motivate animals to run. To verify an elevation in body temperature, rectal temperature was measured using a calibrated thermistor probe (LT-8; Gram Corporation, Saitama, Japan) inserted approximately 6–7 cm past the anal sphincter into the colon before and immediately after exercise. Muscle temperature was also measured immediately after exercise using a needle thermistor probe inserted into one gastrocnemius muscle. After measuring rectal temperature, the animals were anesthetized using isoflurane, and blood glucose and lactate concentrations were measured from the tail vein using the Glutest Neo Super device (Sanwa Kagaku Kenkyusho, Aichi, Japan) and the Lactate Pro device (Arkray, Kyoto, Japan), respectively. Next, blood and gastrocnemius muscles were removed, rapidly frozen in liquid nitrogen, and stored at −80°C until analysis. In this study, the needle-inserted leg was not used in the analyses.

### Heat treatment

To evaluate the effects of body temperature elevation *per se* on signal transduction, HT animals were exposed to single heat treatment (40–41°C for 30 min) in a heat chamber (TVG321AA, Advantec, Tokyo, Japan) without anesthesia, followed by 5 h fasting. Our previous study suggested that this treatment increases rectal temperature from 37–38°C to ~40–41°C within 30 min of initiation [20]. The rectal and muscle temperatures were measured and the animals were sacrificed by the same means described above immediately following heat treatment.

### Measurement of serum insulin concentration

Blood samples were centrifuged at 3,000 rpm for 10 min to obtain serum. Insulin concentration was measured using a commercially available enzyme-linked immunosorbent assay kit (Morinaga Institute of Biological Science, Inc., Kanagawa, Japan) according to the manufacturer’s instructions.

### Sample preparation and immunoblot analysis

Gastrocnemius muscles were powdered under liquid nitrogen and homogenized in ice-cold homogenization buffer (20 mM HEPES, pH 7.4, 0.1 mM EDTA, 4 mM EGTA, 10 mM MgCl_2_, and 0.1% Triton X-100) containing Halt^TM^ Protease inhibitor cocktail (Thermo Scientific, Wilmington, CA, USA) and PhosSTOP (Roche, Penzberg, Germany). The homogenates were centrifuged at 900 × g for 5 min at 4°C. The supernatant were then centrifuged at 12,000 × g for 15 min at 4°C. Protein concentrations in the supernatants were determined using a BCA protein assay kit (Thermo, Rockford, IL, USA). Protein extracts were solubilized in sample buffer (30% glycerol, 5% 2-beta-mercaptethanol, 2.3% SDS, 62.5 mM Tris-HCl pH 6.8, and 0.05% bromophenol blue) at 1.0 mg/ml and incubated at 95°C for 5 min.

Proteins were loaded onto 10% sodium dodecyl sulfate polyacrylamide gel electrophoresis (SDS-PAGE) gels and run at 150 V for 50–60 minutes. Proteins were then transferred to polyvinylidene difluoride (PVDF) membranes at 100 V for 1 h. After transfer, membranes were blocked for 1 h at room temperature in PVDF blocking reagent (Toyobo Co. Ltd., Osaka, Japan). After three washes with Tween-Tris-buffered saline (T-TBS; 40 mM Tris-HCl, 300 mM NaCl, and 0.1% Tween 20, pH 7.5), membranes were incubated with the following primary antibodies: phosphorylated Ser473-Akt (1: 2000; Cell Signaling Technology, #9271, Danvers, MA, USA), Thr308-Akt (1: 2000; Cell Signaling Technology, #2965), Akt (1: 2000; Cell Signaling Technology, #4691), phosphorylated Thr642-AS160 (1: 2000; Cell Signaling Technology, #4288), AS160 (1: 2000; Cell Signaling Technology, #2670), phosphorylated Thr172-AMPKα (1: 2000; Cell Signaling Technology, #2535), AMPK (1: 2000; Cell Signaling Technology, #2532), phosphorylated Ser660-TBC1D1 (1: 2000; Cell Signaling Technology, #6928), phosphorylated Thr590-TBC1D1 (1: 2000; Cell Signaling Technology, #6927), TBC1D1 (1: 2000; Cell Signaling Technology, #5929), phosphorylated Ser79-ACC (1: 2000; Cell Signaling Technology, #3661), or ACC (1: 2000; Cell Signaling Technology, #3662) in dilution buffer overnight at 4°C. After several washes in T-TBS, membranes were incubated with anti-rabbit horseradish peroxidase-conjugated secondary antibodies (1: 10,000; Cell Signaling Technology, #7074) in dilution buffer for 1 h at room temperature. After several washes, bands were visualized using ECL Prime reagents (GE Healthcare, Piscataway, NJ, USA) and the signal was recorded with a ChemiDoc^TM^ Touch Imaging System (Bio-Rad Laboratories, Hercules, CA, USA). Analyses were performed using Image Lab ver. 5.2.1 software (Bio-Rad). Protein phosphorylation was calculated as the ratio of phosphorylated to total protein expression and is expressed using arbitrary units.

### Statistical analysis

Values are expressed as the mean ± standard error. Statistical significance was determined using one-way or two-way analysis of variance (ANOVA). When the ANOVA was significant, group differences were determined using Bonferroni’s *post hoc* test. *P* values *< 0*.*05* were considered statistically significant. All statistical analyses were performed using PRISM v.6.0 software (GraphPad Software, San Diego, CA, USA).

## Results

### Rectal and muscle temperatures

**Fig 1:**
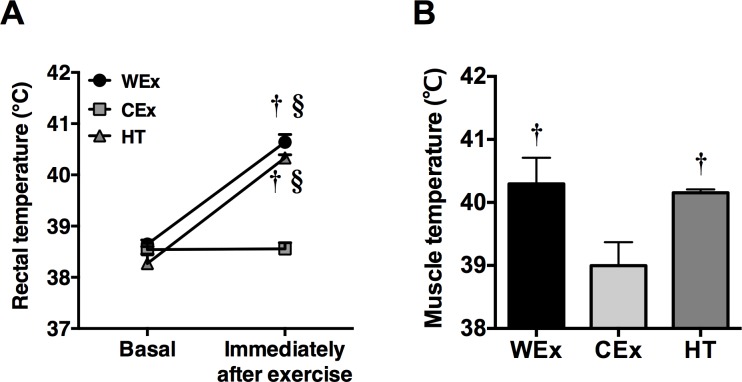
Changes in rectal and muscle temperatures after a single bout of exercise and heat treatment. WEx, *n* = 8 (*black bar*); CEx, *n* = 8 (*gray bar*); HT, *n* = 6 (*dark gray bar*). Values are shown as the means ± standard error (SE). †*p* < 0.05 vs. CEx, §*p* < 0.05 vs. basal values in each group.

The rectal temperature of rats in the WEx group was significantly elevated from 38.68 ± 0.19°C to 40.67 ± 0.47°C immediately after exercise (*p* < 0.05, Fig 1A). In contrast, the elevation of rectal temperature by exercise was completely suppressed in rats in the CEx group (basal: 38.49 ± 0.19°C, after exercise: 38.62 ± 0.61°C). In addition, heat treatment significantly elevated the rectal temperature to similar levels observed in the WEx group (basal: 38.16 ± 0.63°C, after heat treatment: 40.37 ± 0.25°C). The muscle temperatures of rats in the WEx and HT groups were also significantly higher than those of rats in the CEx group (*p* < 0.05, Fig 1B).

### Glucose, insulin and lactate concentration

**Fig 2:**
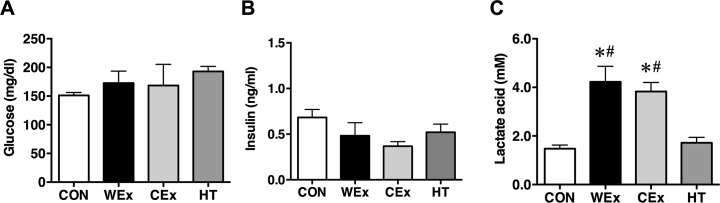
**Serum concentration levels of glucose (A), insulin (B), and lactate (C) after a single bout of exercise and heat treatment.** CON, *n* = 6 (*white bar*); WEx, *n* = 7 (*black bar*); CEx, *n* = 7 (*gray bar*); HT, *n* = 6 (*dark gray bar*). Values are shown as the means ± SE. *p < 0.05 vs. CON, #*p* < 0.05 vs. HT.

The glucose and insulin concentrations immediately after exercise and heat treatment did not differ significantly among the groups (Fig 2A and 2B). The lactate acid concentrations in rats in the WEx and CEx groups immediately after exercise were significantly increased compared with those of rats in the CON group (*p* < 0.05, Fig 2C). However, the lactate concentration did not differ significantly between rats in the WEx and CEx groups. Heat treatment did not increase lactate concentration.

### Phosphorylation of Akt and its downstream signaling molecules

**Fig 3:**
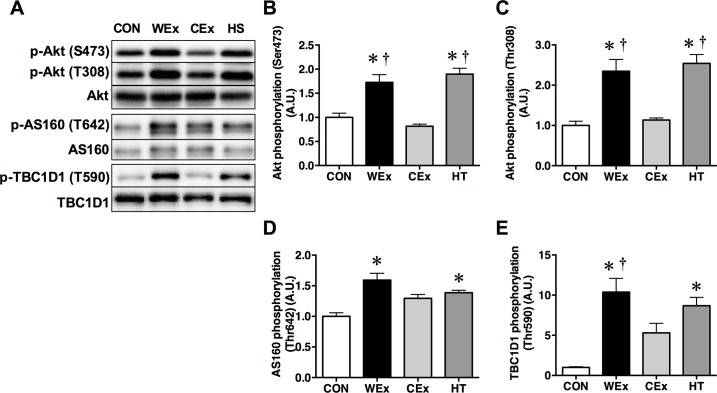
**Representative western blots (A) and the phosphorylation ratio of Akt at Ser473 and Thr308 (B and C), AS160 at Thr642 (D), and TBC1D1 at Thr590 (E) in the gastrocnemius muscle after a single bout of exercise and heat treatment.** CON, *n* = 6 (*white bar*); WEx, *n* = 7 (*black bar*); CEx, *n* = 7 (*gray bar*); HT, *n* = 6 (*dark gray bar*). Values are shown as the means ± SE. *p < 0.05 vs. CON, †*p* < 0.05 vs. CEx.

Phosphorylation of Akt at Thr308 and Ser473 was significantly increased in the muscle of rats in the WEx and HT groups compared to the CON and CEx groups (*p* < 0.05, Fig 3A–3C). However, the phosphorylation of Akt in the muscle of rats in the CON and CEx groups did not differ significantly.

Phosphorylation of AS160 at Thr642 was significantly increased in rats in the WEx and HT groups compared with those in the CON group (*p* < 0.05, Fig 3A and 3D). In addition, the phosphorylation of TBC1D1 at Thr590, an Akt-dependent phosphorylation site, was significantly increased in rats in the WEx and HT groups but not those in the CEx group, compared with the CON group (*p* < 0.05, Fig 3A and 3E).

### Phosphorylation of AMPK and its downstream signaling molecules

**Fig 4:**
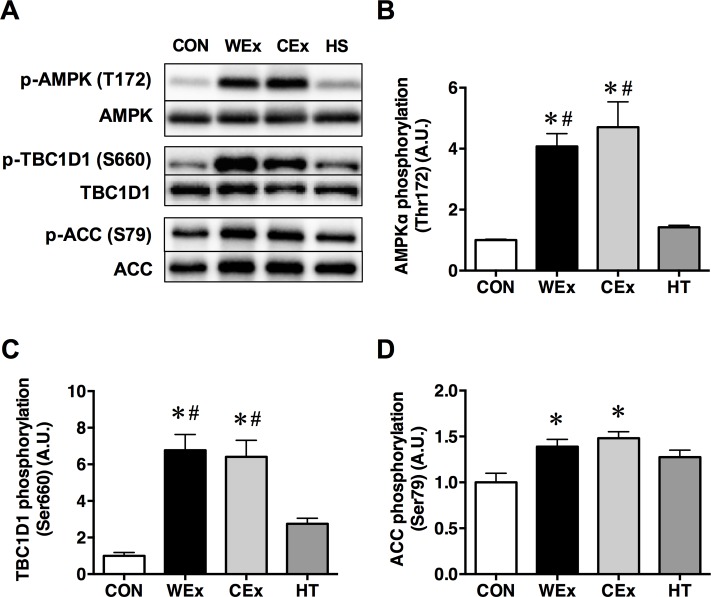
**Representative western blots (A) and the phosphorylation ratio of AMPK at Thr172 (B), TBC1D1 at Ser660 (C), and ACC at Ser79 (D) in the gastrocnemius muscle after a single bout of exercise and heat treatment.** CON, *n* = 6 (*white bar*); WEx, *n* = 7 (*black bar*); CEx, *n* = 7 (*gray bar*); HT, *n* = 6 (*dark gray bar*). Values are shown as the means ± SE. *p < 0.05 vs. CON, #*p* < 0.05 vs. HT.

Phosphorylation of AMPK at Thr172 after exercise was significantly increased in the muscle of rats in the WEx and CEx groups compared with the CON and HT groups (*p* < 0.05, Fig 4A and 4B). In addition, phosphorylation of TBC1D1 at Ser660, an AMPK-dependent phosphorylation site, was increased significantly in the muscle of rats in the WEx and CEx groups compared with the CON and HT groups (*p* < 0.05, Fig 4A and 4C). Phosphorylation of ACC at Ser79 was also increased significantly in the muscle of rats in the WEx and CEx groups compared with the CON group (*p* < 0.05, Fig 4A and 4D). There were no significant differences in the AMPK, TBC1D1, and ACC phosphorylation levels between rats in the WEx and CEx groups.

## Discussion

We reported previously that exercise training without body temperature elevation blunts improvements in insulin resistance in type 2 diabetic rats [4]; however, the underlying mechanisms were unknown. Here, we show that the activation of Akt signaling in skeletal muscle was inhibited by suppression of body temperature elevation during exercise in type 2 diabetic rats. In addition, a single bout of exercise activated AMPK signaling, irrespective of body temperature. To our knowledge, this is the first report that suppression of body temperature elevation during exercise does not activate Akt signaling in the skeletal muscle of type 2 diabetic rats. Moreover, body temperature elevation with and without exercise can activate Akt signaling. Thus, body temperature elevation during exercise may be important for improving insulin resistance due to temperature-dependent Akt activation in skeletal muscle.

Akt is a key player in the metabolic actions of insulin, including glucose transport and synthesis of glycogen and protein; it is impaired in rodent models of, and patients with, type 2 diabetes [21]. To date, two mechanisms of physical exercise-induced Akt activation have been postulated. First, it was suggested that the exercise-induced increase in Akt phosphorylation is due to an increased insulin concentration [22], because IRS-1 [23] and PI3 kinase activity [24] is not increased by exercise or contractile activity. However, Akt phosphorylation was increased after exercise under warm temperature, irrespective of the non-significant differences in serum insulin concentration among the groups examined in this study. Second, exercise and/or muscle contraction *per se* may activate Akt in the skeletal muscle [12] by some as-yet-unknown mechanism. These studies, however, did not consider body temperature elevation during exercise. The Akt phosphorylation status is highly temperature-sensitive and so temperature alterations during exercise may be important factors for activation of Akt and its downstream targets [15, 16]. Here, we used the same protocol as in our previous study to examine the effects of changes in body temperature during exercise on Akt signaling in skeletal muscle [4]. The rectal and muscle temperatures of rats exercised under warm temperature were elevated to approximately 40.5°C, and phosphorylation of Akt and its downstream targets, AS160 and TBC1D1 at Thr590 was increased in skeletal muscle. This temperature elevation was sufficient to increase phosphorylation of Akt and its downstream targets, AS160 and TBC1D1 at Thr590. Importantly, body temperature elevation by heat treatment (without exercise) increased the phosphorylation levels in Akt signaling to similar levels as exercise under warm temperature. Moreover, the additive and/or synergistic effects of body temperature elevation and other factors related to exercise (i.e., muscle contraction and energy expenditure) on Akt phosphorylation were not observed. In contrast, the exercise-induced increase in body temperature was absent under cold temperature, as reported previously [4], and this prevented the increase in Akt phosphorylation. Therefore, an increase in body temperature is essential for activating Akt signaling in the skeletal muscle of type 2 diabetic rats.

A single bout of exercise increased the phosphorylation levels of AMPK and its downstream target, TBC1D1 Ser660 and ACC, irrespective of changes in body temperature. AMPK is an energy-sensing enzyme and its role in muscle glucose transport in response to changes in cellular energy status has been investigated [6]. The AMP/ATP ratio increases rapidly during exercise, causing robust activation of AMPK by Thr172 phosphorylation via upstream kinases and allosteric modification. Activation of AMPK is positively correlated with an increase in muscle glucose uptake in an insulin-independent manner [25]. The active form of AMPK phosphorylates TBC1D1, which may be critical for GLUT4 translocation [26]. In addition, AMPK directly phosphorylates ACC, stimulating fatty-acid oxidation in response to energy demand [27]. ACC was one of the first substrates of AMPK to be identified and is commonly used as a marker of in vivo AMPK activity [28]. AMPK activation and ACC inactivation during short-term exercise is dependent on exercise intensity [27]. In the present study, the lactate concentrations in rats exercised under warm or cold temperature were similar. AMPK phosphorylation levels were also similar between the exercise conditions. Thus, the similar levels of lactate concentration and AMPK phosphorylation under both exercise conditions may partly explain why the exercise intensity in both exercise conditions was comparable. Moreover, body temperature elevation by heat treatment did not increase the AMPK phosphorylation. Therefore, these results suggest that body temperature elevation is not associated with AMPK activation during exercise. In contrast, previous studies have reported that the level of AMPK phosphorylation was increased following acute exposure to heat stress [29, 30], suggesting that elevation of body temperature *per se* activates AMPK in skeletal muscle. Indeed, previous studies have shown that ATP and phosphocreatine levels were decreased by heat stimulation and may have contributed to the activation of AMPK [29]. However, it is believed that physical exercise consumes more ATP than heat stimulation. Thus, it is hypothesized that energetic changes induced by exercise (e.g., muscle contraction) strongly affect the AMPK activation compared with those induced by body temperature elevation.

This study has one major limitation. We were unable to measure the rate of glucose uptake into skeletal muscle. We speculate that exercise under cold conditions may partly attenuate the increased glucose uptake into muscle due to the lack of Akt activation by suppressing body temperature elevation, because previous study revealed that the rate of glucose uptake through the two different mechanisms, insulin signaling and exercise-induced signaling, is additive [31]. Therefore, further studies are required to address this issue.

## Conclusions

In conclusion, we suggest that the differences in body temperature during exercise affect the activation of Akt signaling, but not AMPK signaling, in the skeletal muscle of obese/type 2 diabetic rats. It is possible that body temperature elevation during exercise contributes to improved insulin resistance by activating Akt signaling in skeletal muscle. We propose that the combination of body temperature elevation and exercise may be an effective strategy for glucose maintenance in type 2 diabetic individuals by concurrently stimulating the Akt and AMPK pathways.

## References

[1] DeFronzo RA, Ferrannini E, Sato Y, Felig P, Wahren J. Synergistic interaction between exercise and insulin on peripheral glucose uptake. The Journal of clinical investigation. 1981;68(6):1468–74. 10.1172/JCI110399 ; PubMed Central PMCID: PMCPMC370949. 7033285 PMC370949

[2] Zierath JR, Krook A, Wallberg-Henriksson H. Insulin action and insulin resistance in human skeletal muscle. Diabetologia. 2000;43(7):821–35. 10.1007/s001250051457 . 10952453

[3] Di Meo S, Iossa S, Venditti P. Improvement of obesity-linked skeletal muscle insulin resistance by strength and endurance training. The Journal of endocrinology. 2017;234(3):R159–R81. 10.1530/JOE-17-0186 . 28778962

[4] Tsuzuki T, Kobayashi H, Yoshihara T, Kakigi R, Ichinoseki-Sekine N, Naito H. Attenuation of exercise-induced heat shock protein 72 expression blunts improvements in whole-body insulin resistance in rats with type 2 diabetes. Cell Stress Chaperones. 2017;22(2):263–9. 10.1007/s12192-017-0767-z ; PubMed Central PMCID: PMCPMC5352600. 28155127 PMC5352600

[5] Goodyear LJ, Kahn BB. Exercise, glucose transport, and insulin sensitivity. Annual review of medicine. 1998;49:235–61. Epub 1998/03/24. 10.1146/annurev.med.49.1.235 . 9509261

[6] Rockl KS, Witczak CA, Goodyear LJ. Signaling mechanisms in skeletal muscle: acute responses and chronic adaptations to exercise. IUBMB Life. 2008;60(3):145–53. Epub 2008/04/02. 10.1002/iub.21 ; PubMed Central PMCID: PMC2885767. 18380005 PMC2885767

[7] Witczak CA, Fujii N, Hirshman MF, Goodyear LJ. Ca2+/calmodulin-dependent protein kinase kinase-alpha regulates skeletal muscle glucose uptake independent of AMP-activated protein kinase and Akt activation. Diabetes. 2007;56(5):1403–9. Epub 2007/02/09. 10.2337/db06-1230 . 17287469

[8] Wright DC, Hucker KA, Holloszy JO, Han DH. Ca2+ and AMPK both mediate stimulation of glucose transport by muscle contractions. Diabetes. 2004;53(2):330–5. Epub 2004/01/30. . 14747282 10.2337/diabetes.53.2.330

[9] Kramer HF, Witczak CA, Fujii N, Jessen N, Taylor EB, Arnolds DE, et al Distinct signals regulate AS160 phosphorylation in response to insulin, AICAR, and contraction in mouse skeletal muscle. Diabetes. 2006;55(7):2067–76. 10.2337/db06-0150 . 16804077

[10] Thong FS, Dugani CB, Klip A. Turning signals on and off: GLUT4 traffic in the insulin-signaling highway. Physiology (Bethesda). 2005;20:271–84. 10.1152/physiol.00017.2005 . 16024515

[11] Sakamoto K, Hirshman MF, Aschenbach WG, Goodyear LJ. Contraction regulation of Akt in rat skeletal muscle. The Journal of biological chemistry. 2002;277(14):11910–7. 10.1074/jbc.M112410200 . 11809761

[12] Sakamoto K, Aschenbach WG, Hirshman MF, Goodyear LJ. Akt signaling in skeletal muscle: regulation by exercise and passive stretch. American journal of physiology Endocrinology and metabolism. 2003;285(5):E1081–8. 10.1152/ajpendo.00228.2003 . 12837666

[13] Brozinick JT Jr., Birnbaum MJ. Insulin, but not contraction, activates Akt/PKB in isolated rat skeletal muscle. The Journal of biological chemistry. 1998;273(24):14679–82. . 9614064 10.1074/jbc.273.24.14679

[14] Sherwood DJ, Dufresne SD, Markuns JF, Cheatham B, Moller DE, Aronson D, et al Differential regulation of MAP kinase, p70(S6K), and Akt by contraction and insulin in rat skeletal muscle. The American journal of physiology. 1999;276(5 Pt 1):E870–8. . 10329981 10.1152/ajpendo.1999.276.5.E870

[15] Oehler-Janne C, von Bueren AO, Vuong V, Hollenstein A, Grotzer MA, Pruschy M. Temperature sensitivity of phospho-Ser(473)-PKB/AKT. Biochemical and biophysical research communications. 2008;375(3):399–404. 10.1016/j.bbrc.2008.08.035 . 18721797

[16] Yoshihara T, Naito H, Kakigi R, Ichinoseki-Sekine N, Ogura Y, Sugiura T, et al Heat stress activates the Akt/mTOR signalling pathway in rat skeletal muscle. Acta Physiol (Oxf). 2013;207(2):416–26. 10.1111/apha.12040 . 23167446

[17] Bruss MD, Arias EB, Lienhard GE, Cartee GD. Increased phosphorylation of Akt substrate of 160 kDa (AS160) in rat skeletal muscle in response to insulin or contractile activity. Diabetes. 2005;54(1):41–50. . 15616009 10.2337/diabetes.54.1.41

[18] Taylor EB, An D, Kramer HF, Yu H, Fujii NL, Roeckl KS, et al Discovery of TBC1D1 as an insulin-, AICAR-, and contraction-stimulated signaling nexus in mouse skeletal muscle. The Journal of biological chemistry. 2008;283(15):9787–96. 10.1074/jbc.M708839200 ; PubMed Central PMCID: PMCPMC2442306. 18276596 PMC2442306

[19] Kawano K, Hirashima T, Mori S, Saitoh Y, Kurosumi M, Natori T. Spontaneous long-term hyperglycemic rat with diabetic complications. Otsuka Long-Evans Tokushima Fatty (OLETF) strain. Diabetes. 1992;41(11):1422–8. Epub 1992/11/01. 1397718 10.2337/diab.41.11.1422

[20] Yoshihara T, Ichinoseki-Sekine N, Kakigi R, Tsuzuki T, Sugiura T, Powers S, et al Repeated exposure to heat stress results in a diaphragm phenotype that resists ventilator-induced diaphragm dysfunction. J Appl Physiol (1985). 2015;119(9):1023–31. 10.1152/japplphysiol.00438.2015 26384411

[21] Zierath JR, Krook A, Wallberg-Henriksson H. Insulin action in skeletal muscle from patients with NIDDM. Molecular and cellular biochemistry. 1998;182(1–2):153–60. . 9609124

[22] Esbjornsson M, Rundqvist HC, Mascher H, Osterlund T, Rooyackers O, Blomstrand E, et al Sprint exercise enhances skeletal muscle p70S6k phosphorylation and more so in women than in men. Acta Physiol (Oxf). 2012;205(3):411–22. 10.1111/j.1748-1716.2012.02404.x . 22268492

[23] Zhou Q, Dohm GL. Treadmill running increases phosphatidylinostol 3-kinase activity in rat skeletal muscle. Biochemical and biophysical research communications. 1997;236(3):647–50. 10.1006/bbrc.1997.7028 . 9245706

[24] Goodyear LJ, Giorgino F, Balon TW, Condorelli G, Smith RJ. Effects of contractile activity on tyrosine phosphoproteins and PI 3-kinase activity in rat skeletal muscle. The American journal of physiology. 1995;268(5 Pt 1):E987–95. 10.1152/ajpendo.1995.268.5.E987 . 7762655

[25] Merrill GF, Kurth EJ, Hardie DG, Winder WW. AICA riboside increases AMP-activated protein kinase, fatty acid oxidation, and glucose uptake in rat muscle. The American journal of physiology. 1997;273(6 Pt 1):E1107–12. . 9435525 10.1152/ajpendo.1997.273.6.E1107

[26] Sakamoto K, Holman GD. Emerging role for AS160/TBC1D4 and TBC1D1 in the regulation of GLUT4 traffic. American journal of physiology Endocrinology and metabolism. 2008;295(1):E29–37. 10.1152/ajpendo.90331.2008 ; PubMed Central PMCID: PMCPMC2493596. 18477703 PMC2493596

[27] Rasmussen BB, Winder WW. Effect of exercise intensity on skeletal muscle malonyl-CoA and acetyl-CoA carboxylase. J Appl Physiol (1985). 1997;83(4):1104–9. . 9338417 10.1152/jappl.1997.83.4.1104

[28] Carling D, Zammit VA, Hardie DG. A common bicyclic protein kinase cascade inactivates the regulatory enzymes of fatty acid and cholesterol biosynthesis. FEBS letters. 1987;223(2):217–22. . 2889619 10.1016/0014-5793(87)80292-2

[29] Koshinaka K, Kawamoto E, Abe N, Toshinai K, Nakazato M, Kawanaka K. Elevation of muscle temperature stimulates muscle glucose uptake in vivo and in vitro. The journal of physiological sciences: JPS. 2013;63(6):409–18. 10.1007/s12576-013-0278-3 . 23836025 PMC10718043

[30] Goto A, Egawa T, Sakon I, Oshima R, Ito K, Serizawa Y, et al Heat stress acutely activates insulin-independent glucose transport and 5'-AMP-activated protein kinase prior to an increase in HSP72 protein in rat skeletal muscle. Physiol Rep. 2015;3(11). doi: 10.14814/phy2.12601 ; PubMed Central PMCID: PMCPMC4632964. 26542263 10.14814/phy2.12601 PMC4632964

[31] Lund S, Holman GD, Schmitz O, Pedersen O. Contraction stimulates translocation of glucose transporter GLUT4 in skeletal muscle through a mechanism distinct from that of insulin. Proceedings of the National Academy of Sciences of the United States of America. 1995;92(13):5817–21. ; PubMed Central PMCID: PMCPMC41592. 7597034 10.1073/pnas.92.13.5817 PMC41592

